# Single-Cell Transcriptome Analysis Reveals Inter-Tumor Heterogeneity in Bilateral Papillary Thyroid Carcinoma

**DOI:** 10.3389/fimmu.2022.840811

**Published:** 2022-04-20

**Authors:** Tiantian Wang, Jinyuan Shi, Luchuan Li, Xiaoming Zhou, Hui Zhang, Xiaofang Zhang, Yong Wang, Lian Liu, Lei Sheng

**Affiliations:** ^1^ Department of Thyroid Surgery, The Second Affiliated Hospital of Zhejiang University College of Medicine, Hangzhou, China; ^2^ Department of Thyroid Surgery, The First Hospital of China Medical University, Shenyang, China; ^3^ Department of Thyroid Surgery, General Surgery, Qilu Hospital of Shandong University, Jinan, China; ^4^ Department of Scientific Research, Shandong Provincial Hospital Affiliated to Shandong First Medical University, Jinan, China; ^5^ Department of Thyroid Surgery, Shandong Provincial Hospital Affiliated to Shandong First Medical University, Jinan, China; ^6^ Department of Pathology, Basic Medical College of Shandong University, Jinan, China; ^7^ Department of Medical Oncology, Qilu Hospital, Cheeloo College of Medicine, Shandong University, Jinan, China

**Keywords:** scRNA-seq, papillary thyroid carcinoma, tumor microenvironment, immune dysfunction, bilaterality

## Abstract

**Background:**

The tumor microenvironment (TME) plays a pivotal role in cancer progression in papillary thyroid carcinoma (PTC), yet the composition and the phenotype of cells within the TME in bilateral PTC are poorly understood.

**Methods:**

We performed unbiased transcriptome-wide single-cell RNA sequencing (scRNA-seq) analysis on 29,561 cells from 3 pairs of bilateral PTC and 1 non-tumor thyroid sample. The results of the analysis were validated by a large-scale bulk transcriptomic dataset deposited in The Cancer Genome Atlas (TCGA) database.

**Results:**

Our integrative analysis of thyroid follicular cells revealed 42 signaling pathways enriched in malignant follicular cells, including cytokine–cytokine receptor interaction, PI3K/Akt signaling pathway, mitogen-activated protein kinase (MAPK) signaling pathway, and tumor necrosis factor (TNF) signaling pathway. A 6-gene signature (*CXCL3*, *CXCL1*, *IL1A*, *CCL5*, *TNFRSF12A*, and *IL18*) in the cytokine–cytokine receptor interaction pathway was constructed to predict the prognosis of patients with PTC, with high risk scores being associated with decreased overall survival [hazard ratio (HR) = 3.863, 95% CI = 2.233−6.682, *p* < 0.001]. Gene set variation analysis (GSVA) indicated that the pathways enriched in bilateral PTC were significantly different, indicating great heterogeneity in bilateral PTC, even with the same *BRAF V600E* mutation. Comprehensive analysis of T cells revealed that the proportion of CD8^+^ tissue-resident memory T cells expressing *IFNG* decreased in tumor samples with advanced N stage. Within the myeloid compartment, the ratio of suppressive M2-like to pro-inflammatory M1-like macrophages increased with advanced disease stage, which was confirmed in the bulk dataset using transcriptomic profiles. In addition, we also identified numerous biologically critical interactions among myeloid cells, T cells, and follicular cells, which were related to T-cell recruitment, M2-like macrophage polarization, malignant follicular cell progression, and T-cell inhibitory signaling.

**Conclusion:**

Our integrative analyses revealed great inter-tumor heterogeneity within the TME in bilateral PTC, which will offer assistance for precise diagnosis and treatment.

## Introduction

Papillary thyroid carcinoma (PTC) is the most common malignant phenotype of thyroid cancer, with a rapidly increasing number of new cases globally (1, 2). Multifocality is a common phenomenon in patients with PTC (3). Multifocal PTC has been associated with increased risk of locoregional lymph node metastasis (3). However, the genetic mechanisms, molecular pathogenesis, management, and prognosis of multifocal PTC remain controversial (4, 5). Approaches to infer the clonality of multifocal PTC have evolved from X-chromosome inactivation (6), determination of loss of heterozygosity (7), and mutational analysis (8) to the recent next-generation sequencing (9). The most common genetic somatic alteration is the mutation of *BRAF* (59.7%) in PTC, followed by *NRAS* (8.5%) and *HRAS* (3.5%), encoding activators in the mitogen-activated protein kinase (MAPK) signaling pathway (10). Two opposite viewpoints, either independent origin or intraglandular dissemination, have been proposed to infer the clonal origin of multifocal PTC based on the profiles of the next-generation sequencing (9). However, such approaches are restricted to genetic diversity and fail to reveal the molecular or transcriptional phenotype of multifocal or bilateral tumors.

Either thyroid lobectomy or total thyroidectomy is a common alternative option for patients with PTC (11). In this context, clearly deciphering the genetic relations among multifocal PTC lesions and assessing the malignant potential of each thyroid nodule could have critical diagnostic, therapeutic, and prognostic implications. In addition, the tumor immune microenvironment (TIME) has also been implicated to play a critical role in cancer progression in several cancers (12, 13), including thyroid cancer (14). Prior effects to characterize the TME of PTC using bulk RNA sequencing have advanced our understanding of how different cell lineages within the TME may affect the prognosis of PTC, but with limited resolution (14). In contrast, single-cell RNA sequencing (scRNA-seq) enables comprehensive characterization of the cellular compositions and transcriptional phenotypes of malignant cells and the surrounding immune cells (13, 15).

Herein, we performed single-cell transcriptomic profiling coupled with genomic DNA sequencing of 3 pairs of bilateral PTC and 1 non-tumor thyroid tissue to decipher the inter-tumor heterogeneity in bilateral PTC, with implications for the diagnosis and prognosis of multifocal PTC.

## Methods and Materials

### Patient Samples

**Figure 1:**
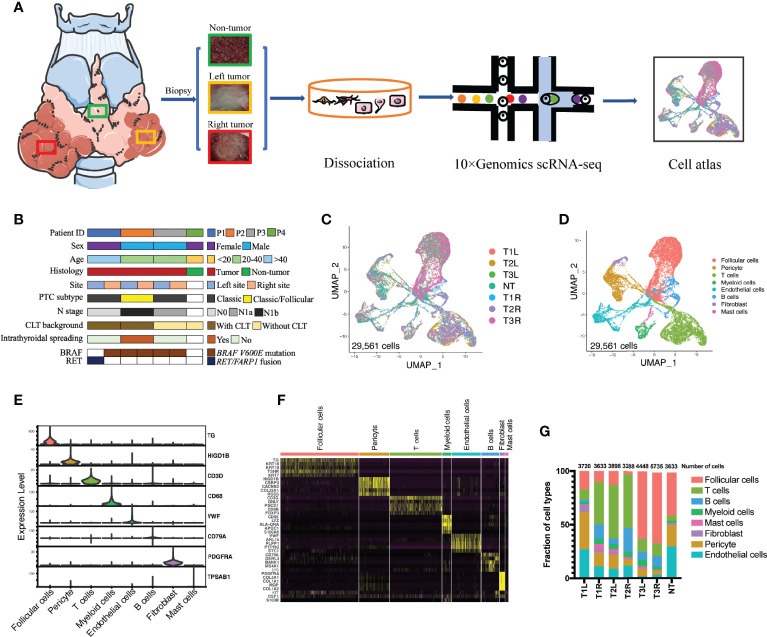
Single-cell transcriptomic profiling of bilateral papillary thyroid carcinoma (PTC). **(A)** Schematic diagram of the single-cell transcriptomic profiling of non-tumor (NT) thyroid tissue (*n* = 1) and bilateral PTC (*n* = 3) across disease stages. **(B)** Clinical and pathological characteristics of the patient cohort with the distribution of mutation events. **(C)** All PTC and NT thyroid tissues were well represented in the dataset. Uniform manifold approximation and projection (UMAP) for dimension reduction colored by tissue origin (total number of cells = 29,561). **(D)** Single-cell RNA sequencing (scRNA-seq) analysis revealed eight known cell lineages (cell populations labeled; total number of cells = 29,561). **(E)** Violin plots showing the smoothed expression distributions of canonical marker genes in the eight cell lineages. **(F)** Heatmap of the top 5 signature genes in each cell lineage. **(G)** Proportion of each cell lineage in seven samples. *T1L* and *T1R*: PTC from the left lobe and right lobe of patient P1, respectively. *T2L* and *T2R*: PTC from the left lobe and right lobe of patient P2, respectively. *T3L* and *T3R*: PTC from the left lobe and right lobe of patient P3, respectively.

All postoperative thyroid specimens were collected either from the Department of Thyroid Surgery, Qilu Hospital of Shandong University or The Second Affiliated Hospital of Zhejiang University, with Institutional Review Board approval and written informed consent obtained from all subjects. Three pairs of tumor samples from patients with bilateral PTC and one non-tumor thyroid tissue as a control were used for viable cell scRNA-seq analysis (**Figure 1A**). The clinical and pathological characteristics of the patients were collected.

### Isolation of Single Cells, Cell Capture, and cDNA Synthesis

All freshly resected thyroid specimens were immediately washed twice with phosphate-buffered saline (PBS) and digested with digestive enzyme mixture containing 10 ml pre-warmed RPMI 1640 (Thermo Fisher Scientific, Waltham, MA, USA), 1 mg/ml type IV collagenase (Sigma, St. Louis, MO, USA), 2 mg/ml dispase (Roche, Basel, Switzerland), and 10 U/µl DNase (Roche) for an hour at 37°C. The reaction was deactivated by adding 10% fetal bovine serum (FBS; Thermo Fisher Scientific). Cell suspensions were filtered using a 70-μm filter and then centrifuged at 500 rpm for 6 min at 4°C to pellet dead cells and red blood cells. The cells were washed twice and suspended in PBS with 0.5% bovine serum albumin (BSA; Sigma). Cell suspensions (300–600 living cells per microliter determined by Count Star) were loaded onto the Chromium Single Cell Controller (10X Genomics, Pleasanton, CA, USA) to generate single-cell gel beads in the emulsion using Single Cell 3′ Library and Gel Bead Kit v3.1 (1000121; 10X Genomics) and Chromium Single Cell G Chip Kit (1000120; 10X Genomics) according to the manufacturer’s protocol. In short, single cells were suspended in PBS containing 0.04% BSA.

About 6,000 cells were added to each channel, and the target cells that will be recovered were estimated to be about 3,000 cells. Captured cells were lysed and the released RNA barcoded through reverse transcription in individual gel beads in emulsion (GEMs). Reverse transcription was performed on a S1000TM Touch Thermal Cycler (Bio-Rad, Hercules, CA, USA) at 53°C for 45 min, followed by 85°C for 5 min, and hold at 4°C. The complementary DNA (cDNA) was generated, amplified, and then the quality assessed using an Agilent 4200 (performed by CapitalBio Technology, Beijing, China).

### Single-Cell RNA-Seq Library Preparation

According to the manufacturer’s introduction, scRNA-seq libraries were constructed using the Single Cell 3′ Library and Gel Bead Kit v3.1. The libraries were finally sequenced using an Illumina Novaseq6000 sequencer with a sequencing depth of at least 100,000 reads per cell with pair-end 150-bp strategy (performed by CapitalBio Technology, Beijing, China).

### Single-Cell Gene Expression Quantification and Cluster Classification

The sequencing data from 10X Genomics were aligned against the human reference genome (hg19). Raw gene expression matrices were processed using the Seurat R package (version 4.0.3). Standard scRNA-seq filtering excludes low-quality cells with less than 201 or over 5,000 expressed genes, or over 25% unique molecular identifiers (UMIs) derived from the mitochondrial genome.

The gene expression matrices of the remaining 29,561 cells were normalized to the total cellular UMI counts. The normalized expression was scaled (scale factor = 1e4) by regressing out the total cellular UMI counts. Highly variable genes were calculated using the Seurat FindVariableGenes function with default parameters. Then, we performed principal component analysis (PCA) using 2,000 of the highly variable genes. The top 30 significant principal components (PCs) were selected to perform uniform manifold approximation and projection (UMAP) dimension reduction, and clusters were determined using the FindClusters function (dims.use = 1:15, resolution = 0.8). The UMAP analysis was used for dimension reduction and visualization of gene expressions. Unbiased clustering generated 32 main clusters that were annotated to 8 known cell types according to canonical marker genes.

### Analysis of Differentially Expressed Genes

To explore the differentially expressed genes (DEGs) in each cluster or between tumor and non-tumor samples, the FindMarkers tool was applied to calculate the DEGs. The results were visualized by a dot plot and a volcano plot. Gene set variation analysis (GSVA) was performed to observe the heterogeneity of the enriched pathways in bilateral thyroid cancer samples from the same patient.

### Pseudotime Trajectory Analysis

The Monocle 2 (http://cole-trapnell-lab.github.io/monocle-release/) R package was used to perform pseudotime trajectory analysis, revealing dynamic changes in the transcriptome of developing a specific cell lineage. The cells were dimensionally reduced by the DDRTree method, sequenced according to pseudotime, and finally ordered to visualize the trajectory (16).

### Cell–Cell Interaction Analysis Using CellPhoneDB

The CellPhoneDB package (www.cellphonedb.org) was adopted to explore the ligand–receptor interactions between the different cell subtypes as previously described (17). We performed analysis using the Python package (https://github.com/Teichlab/cellphonedb) running within the integrated development environment (IDE) PyCharm (version 2021.2, Professional Edition, by JetBrains). The interactions between distinct cell subtypes *via* putative ligand–receptor pairs were visualized by a heatmap and a dot plot.

### DNA Extraction and 21-Gene DNA Sequencing

Genomic DNA was extracted from formalin-fixed and paraffin-embedded (FFPE) thyroid tumor tissues using the QIAamp DNA FFPE Tissue Kit (QIAGEN, Valencia, CA, USA) according to the manufacturer’s instructions. Next-generation sequencing was performed using a hybridization capture-based assay, AmoyDx Thyroid Carcinoma 21-Gene Panel (ADx TC21; Amoy Diagnostics, Xiamen, China), including selected exons and introns from 21 genes (*AKT1*, *ALK*, *BRAF*, *CTNNB1*, *EIF1AX*, *GNAS*, *HRAS*, *KRAS*, *NRAS*, *NTRK1*, *NTRK3*, *PAX8*, *PDGFRA*, *PIK3CA*, *PTEN*, *RASAL1*, *RET*, *TERT*, *TP53*, *TSC2*, and *TSHR*), which can detect single nucleotide variants (SNVs), small insertions and deletions (indels), and gene fusion by the AmoyDx Analyze System.

### Prognostic Validation of Gene Signatures

The least absolute shrinkage and selection operator (LASSO) Cox regression model was utilized to construct a multigene signature of the DEGs in the cytokine–cytokine receptor interaction pathway in The Cancer Genome Atlas (TCGA) PTC cohort (https://portal.gdc.cancer.gov/repository) using the “glmnet” package in R. The patients were stratified into a high risk score and a low risk score group based on the multigene signature. A Kaplan–Meier curve was constructed to visualize the differences in the overall survival (OS) between patients with high and low risk scores.

### Copy Number Variation Analysis

We inferred the copy number variations (CNVs) of all patients with inferCNV (https://github.com/broadinstitute/inferCNV) using the scRNA-seq transcriptomic profiles (18). We used T cells and B cells as baseline to estimate the CNVs of all the remaining cells. Initial CNVs were estimated by sorting the genes based on their genomic locations and averaging the gene expressions as previously described (19). Cell lineages were initially classified by canonic gene markers using the Seurat package. For the 10X Genomics single-cell data, the cutoff value was 0.1.

### Statistical Analysis

Multivariate Cox regression analyses were performed to screen the predictors for OS. A receiver operating characteristic (ROC) curve was used to determine the predictive capability of the signatures. *P*-values <0.05 were considered statistically significant.

## Results

### A Single-Cell Transcriptome Atlas in Malignant and Normal Thyroid Tissues

To investigate the similarities and disparities of the cell populations and the associated molecular characteristics of bilateral PTC, 3 pairs of PTC samples from three patients with bilateral PTC and one non-tumor sample as a control were included in our scRNA-seq performed using the droplet-based 10X Genomics platform (**Figure 1A**). The genetic profiles of these tumors, including the SNVs, indels, and gene fusions, were characterized using DNA sequencing (**Figure 1B**). The bilateral tumors harbored the same *BRAF V600E* point mutation in patients P2 and P3, whereas different types of mutations were found in the left (*RET/FARP1* fusion) and right (*BRAF V600E* point mutation) tumors in patient P1. The clinical characteristics of these participants, including sex, age, and pathological features, were collected at the time of recruitment (**Figure 1B** and **Supplementary Figure S1**). After filtering out low-quality cells, 29,561 cells were retained for further scRNA-seq analysis. After normalization and PCA, we performed graph-based clustering to segregate the cells into 32 clusters, which could be assigned to 8 known cell clusters according to canonical lineage markers (**Figures 1C**–**F**) and DEGs (**Supplementary File 1**): follicular cells (10,266 cells, 34.7%, marked with *TG*, *TSHR*, and *TPO*); B cells (2,347 cells, 7.9%, marked with *CD79A*); T cells (6,907 cells, 23.4%, marked with *CD3D*, *CTLA4*, and *CD8B*); myeloid cells (1179 cells, 4.0%, marked with *CD68* and *CD14*); mast cells (415 cells, 1.4%, marked with *TPSAB1*); fibroblasts (670 cells, 2.3%, marked with *PDGFRA*); endothelial cells (3,813 cells, 12.9%, marked with *VWF*); and pericytes (3,964 cells, 13.4%, marked with *HIGD1B*). Differences in the proportions of each cell type in bilateral tumors with different genetic backgrounds [T1L (tumor from the left lobe in patient 1) *vs.* T1R (tumor from the right lobe in patient 1)] were more obvious than those in tumors with the same *BRAF V600E* mutation [T2L (tumor from the left lobe in patient 2) *vs.* T2R (tumor from the right lobe in patient 2) and T3L (tumor from the left lobe in patient 3) *vs.* T3R (tumor from the right lobe in patient 3)] (**Figure 1G**).

### Enriched Pathways in Malignant Thyroid Follicular Cells

**Figure 2:**
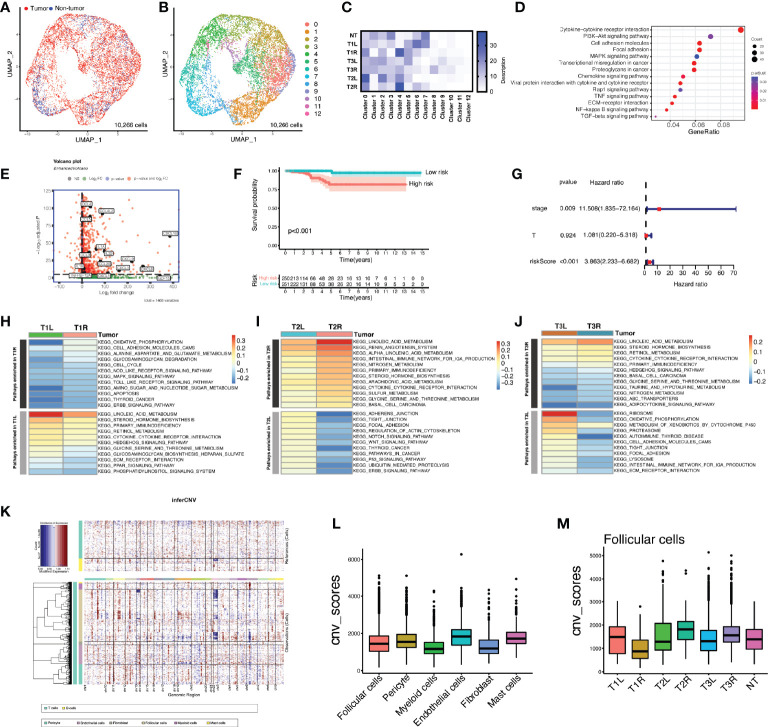
Heterogeneity of the enriched pathways and copy number variation (CNV) in bilateral malignant thyroid follicular cells. **(A)** Uniform manifold approximation and projection (UMAP) plot of the classification of 10,266 malignant and non-malignant thyroid follicular cells. **(B)** UMAP plot of 13 clusters of thyroid follicular cells (*n* = 10,266). **(C)** Heatmap of the proportions of 13 clusters in each sample. *T1L* and *T1R*: papillary thyroid carcinoma (PTC) from the left lobe and right lobe of patient P1, respectively. *T2L* and *T2R*: PTC from the left lobe and right lobe of patient P2, respectively. *T3L* and *T3R*: PTC from the left lobe and right lobe of patient P3, respectively. **(D)** Dot plot showing Kyoto Encyclopedia of Genes and Genomes (KEGG) pathways enriched in malignant thyroid follicular cells compared with non-malignant cells. **(E)** Volcano plot depicting the representation of overexpressed signature genes of the cytokine–cytokine receptor interaction pathway in malignant thyroid follicular cells. **(F)** Prognostic value of the risk score calculated based on signature genes of the cytokine–cytokine receptor interaction pathway in The Cancer Genome Atlas (TCGA) cohort of patients with papillary thyroid carcinoma. **(G)** Kaplan–Meier curve of the overall survival of patients from TCGA PTC cohort grouped by low and high risk scores of the 6-gene signature. **(H–J)** Gene set variation analysis (GSVA) showing great heterogeneity of the enriched pathways in bilateral thyroid cancer samples from the same patient. **(K)** Heatmap of the CNV profiles of all cell subtypes of patients with bilateral PTC. Red indicates genomic copy number gains and blue indicates copy number losses. The *x*-axis shows all chromosomes, while the *y*-axis is marked by cell subtypes within the tumor microenvironment. **(L)** Comparison of the CNV scores among different cell subtypes. **(M)** Comparison of the CNV scores in follicular cells of each sample.

Unsupervised clustering revealed that thyroid follicular cells were classified into 13 distinctive clusters in tumor and non-tumor thyroid tissues (**Figures 2A**–**C**). Non-tumor follicular cells were mainly enriched in clusters 0–3 and clusters 6 and 7, whereas clusters 4 and 5 and clusters 8–12 were largely unique to malignant thyroid follicular cells. To decipher the molecular disparity of malignant and non-malignant thyroid follicular cells, we performed Kyoto Encyclopedia of Genes and Genomes (KEGG) pathway analysis. The full lists of DEGs were compared between tumorous and non-tumorous follicular cells (**Supplementary File 2**). Compared with non-malignant thyroid follicular cells, malignant thyroid follicular cells were enriched in cytokine–cytokine receptor interaction, PI3K/Akt signaling pathway, MAPK signaling pathway, tumor necrosis factor (TNF) signaling pathway, focal adhesion, proteoglycans in cancer, chemokine signaling pathway, transcriptional misregulation in cancer, and cell adhesion molecules (**Figure 2D** and **Supplementary Table S1**). LASSO Cox regression analysis was utilized based on the 44 genes in the cytokine–cytokine receptor interaction pathway (**Figure 2E** and **Supplementary Table S2**). The DEGs were also analyzed patient by patient between the right and left tumors (**Supplementary Figure S2**), indicating heterogeneity in bilateral PTC. A 6-gene signature (*CXCL3*, *CXCL1*, *IL1A*, *CCL5*, *TNFRSF12A*, and *IL18*) was constructed to predict the prognosis of patients with PTC. The risk score for each patient was calculated using the following formula: risk score = *CXCL3* × 1.22 + *CXCL1* × 0.38 + *IL1A* × 0.69 + *CCL5* × (−0.13) + *TNFRSF12A* × (−0.41) + *IL18* × (−0.65). The Kaplan–Meier survival curves indicated that patients with higher risk scores displayed significantly worse OS compared to patients with lower risk scores (**Figure 2F**). Multivariate Cox regression analysis indicated that the risk score of the 6-gene signature was an independent prognostic predictor for OS [hazard ratio (HR) = 3.863, 95% CI = 2.233−6.682, *p* < 0.001] (**Figure 2G**). The ROC curve analysis indicated that the predictive capability of the 6-gene signature for the prognosis of patients with PTC was particularly high, with area under the curve (AUC) values of 0.997 for 1-year OS, 0.810 for 3-year OS, and 0.821 for 5-year OS (**Supplementary Figure S3**). GSVA indicated that the enriched pathways were significantly different in bilateral PTC samples from the same patient (**Figures 2H**–**J**), indicating great heterogeneity in bilateral PTC, even with the same *BRAF V600E* mutation. Subsequently, we inferred the CNVs in the different cell subtypes (**Figure 2K**). The analyses showed that follicular cells were mainly involved in the copy number gains of chromosomes 1 and 12 and the copy number loss of chromosome 22 (**Figure 2K**). However, the overall CNV scores of follicular cells were similar to those of other stromal and immune cells, consistent with a phenotype of low mutational burden in thyroid cancer compared with normal cells (**Figure 2L**) (20). Furthermore, the inferred CNVs in the 3 pairs of bilateral PTC samples showed both intra- and inter-patient heterogeneity (**Figure 2M** and **Supplementary Figure S4**), in particular in malignant thyroid follicular cells with different genetic mutations.

### Transcriptional Heterogeneity in Tumor-Infiltrating T Lymphocytes

**Figure 3:**
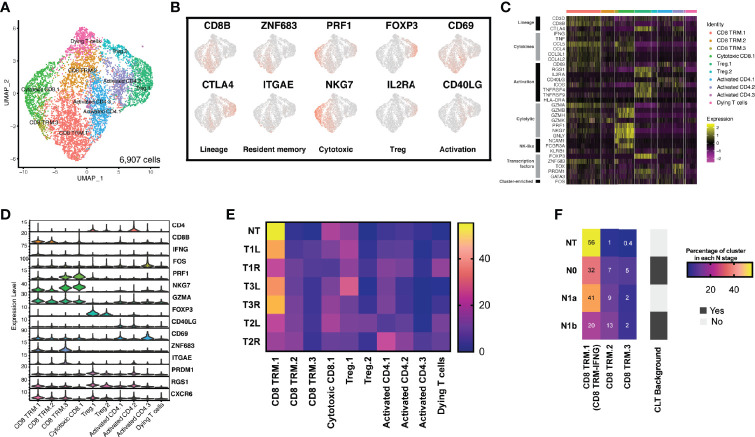
The T-cell landscape of papillary thyroid carcinoma reveals transcriptionally heterogeneous cell populations. **(A)** Sub-clustering of T cells (*n* = 6,907) showing transcriptional heterogeneity with both CD8^+^ and CD4^+^ T cells. Major groups of T-cell lineages labeled. **(B)** Uniform manifold approximation and projection (UMAP) feature plot representation of marker gene expressions with individually identified T-cell populations and phenotypic states. **(C)** Heatmap of T-cell lineages and functional markers providing phenotypic information for individual T-cell populations. **(D)** Violin plots showing the expression distributions of canonical marker genes in T-cell lineages. **(E)** Different T-cell populations were enriched in different thyroid samples. Heatmap representation of the proportion of each T-cell cluster from each sample. **(F)** CD8 TRM-IFNG T cells were enriched in non-tumor (NT) thyroid tissue, CLT, chronic lymphocytic thyroiditis.

To better under the transcriptional heterogeneity within tumor-infiltrating T lymphocytes (TILs), we identified T cell clusters expressing the known T-cell marker (*CD3D*). With graph-based clustering on this subset of 6,907 T cells, 10 individual T-cell populations were identified, falling into four broad categories (**Figure 3A**). These included CD8^+^ tissue-resident memory (TRM) T cells, cytotoxic CD8^+^ T cells, CD4^+^ T cells with activated phenotypes, and regulatory T cells (Tregs).

We explored the characteristics of each T-cell population by differential gene expression analysis of each cluster compared with all other T cells and by assessing the expressions of a panel of genes associated with T-cell lineage. Among the CD4^+^ T cells, two broad groups were identified. The first group (two clusters) expressed high levels of *IL2RA*/CD25 and *FOXP3*, consistent with a Treg population (designated as Treg.1 and Treg.2) (**Figures 3B**–**D**). The second group (three clusters: activated CD4.1, activated CD4.2, and activated CD4.3) expressed *CD40LG*/CD40L and *CD69*, consistent with an activated CD4^+^ T phenotype.

One CD8^+^ T-cell cluster expressed cytotoxic molecules (*PRF1* and *GZMA*) and high levels of *NKG7*, *PRDM1*, and *ID2*, consistent with a cytotoxic population (21). This cluster expressed high levels of cytotoxic genes (*GZMA* and *PRF1*) and markers of both CD8^+^ T cells (*CD3D* and *CD8B*) and natural killer (NK) cells (*KLRB1*, *NCAM1*/CD56, and *FCGR3A*/CD16), indicating some mixtures of NK cells or CD8^+^ NK-like cells within this cluster. Three CD8^+^ T-cell clusters (CD8 TRM.1, CD8 TRM.2, and CD8 TRM.3) expressed *ZNF683*/Hobit, *ITGAE*/CD103, *PRDM1*, *CD69*, *RGS1*, and *CXCR6* (**Figures 3B–D**), consistent with a tissue-resident memory phenotype (22). Finally, one additional T-cell cluster was identified with relatively low expressions of the most described gene markers, most consistent with a dying T-cell population.

We assessed the proportion of each cluster after normalizing for the total number of T cells per sample (**Figure 3E**). Only CD8 TRM.1 T cells changed significantly across the different samples, while the proportions of all the other T cells remained conservative across the different samples. We further found that CD8 TRM.1 expressed higher levels of *IFNG* (termed as CD8 TRM-IFNG). We assessed the composition of CD8 TRM-IFNG, CD8 TRM.2, and CD8 TRM.3 in each cluster with respect to the local lymph node stage (N stage). We found that the proportion of CD8 TRM-IFNG T cells decreased in tumor samples with advanced N stage, particularly in samples with a background of chronic lymphocytic thyroiditis, indicative of high T-cell infiltration (**Figure 3F**). Moreover, the proportion of CD8 TRM-IFNG was more heterogeneous in bilateral tumors (T1L *vs.* T1R) harboring different types of mutations than in paired samples with the same *BRAF V600E* mutation (T2L *vs.* T2R and T3L *vs.* T3R) (**Figure 3E**).

### Increased Ratio of M2-Like/M1-Like Macrophages in Advanced Disease

**Figure 4:**
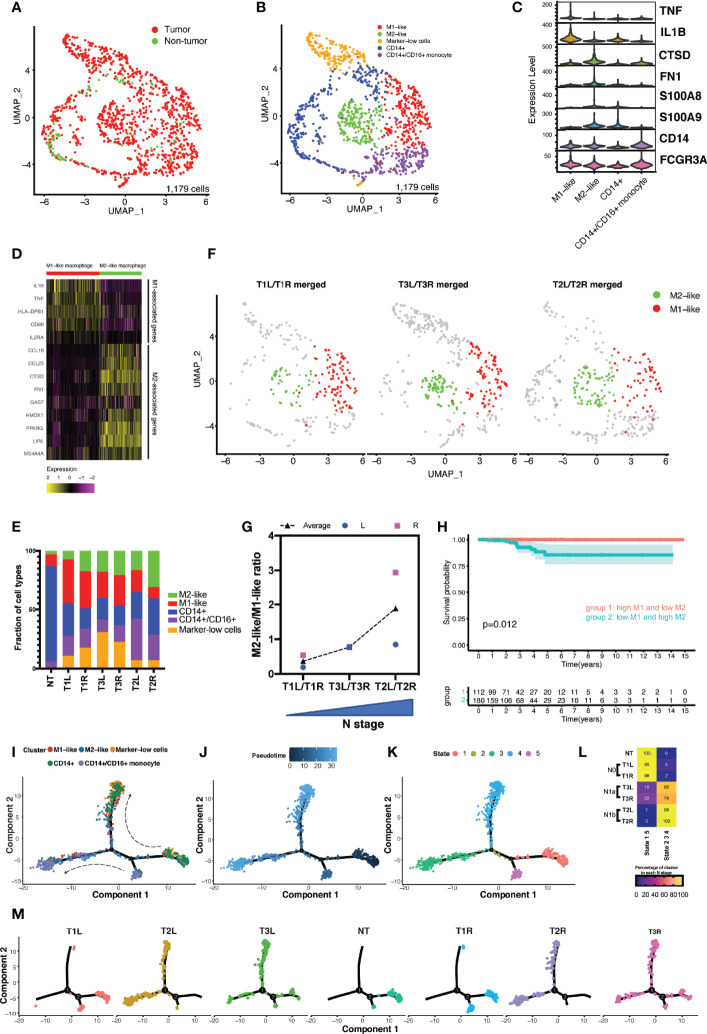
Analysis of the myeloid landscape revealed increased ratio of M2-like/M1-like macrophages in more advanced thyroid cancer. **(A)** Uniform manifold approximation and projection (UMAP) plot of the classification of myeloid cells (*n* = 1,179) from malignant and non-malignant thyroid tissues. **(B)** Sub-clustering of myeloid cells (*n* = 1,179) identified CD14^+^ monocytes (*n* = 307), CD14^+^/CD16^+^ monocytes (*n* = 205), M1-like macrophages (*n* = 257), M2-like macrophages (*n* = 200), and marker-low cells (*n* = 187). **(C)** Violin plot showing the expression distributions of canonical marker genes in four cell lineages. **(D)** M1-like and M2-like signature genes differentially expressed in M1-like and M2-like macrophages. **(E)** Fractions of cell types of myeloid cells in different thyroid samples. **(F)** M1-like and M2-like cells highlighted in the UMAP plot of the merged bilateral samples from the same patient. **(G)** Increased ratio of M2-like/M1-like macrophage was associated with the increased stage of local lymph node metastasis. **(H)** Kaplan–Meier curve of the overall survival of patients from The Cancer Genome Atlas (TCGA) papillary thyroid carcinoma (PTC) cohort grouped by levels of M1-like and M2-like macrophages. **(I–K)** Developmental trajectories of myeloid cells displayed by clusters **(I)** and ordered by pseudotime **(J)** and states **(K)**. **(L)** Association of the proportions of myeloid cells in different trajectory states with disease stage. *N0*, no lymph node metastasis; *N1a*, with central lymph node metastasis; *N1b*, with lateral lymph node metastasis. **(M)** Developmental trajectory of myeloid cells in each sample.

Myeloid cells play a pivotal role in tumor initiation and progression in the tumor microenvironment (TME) (12, 23). We performed graph-based clustering on the subset of 1,179 myeloid cells (excluding mast cells) in tumor and non-tumor thyroid samples, which revealed seven individual cell populations with an unsupervised clustering approach (**Supplementary Figures S5A, B**) falling into five main categories (**Figures 4A, B**). Cell types were identified based on the DEGs and known lineage markers (**Figure 4C**). A total of 365 DEGs (log2FC > 1 and *p*_val_adj < 0.01) were identified in myeloid cells between tumorous and non-tumorous samples (**Supplementary File 3**). A 4-gene signature was constructed, and a high risk score of the 4-gene signature was shown to be an independent prognostic predictor for OS in patients with PTC (HR = 4.607, 95% CI = 2.432−8.729, *p* < 0.001) (**Supplementary Figures S5D**–**I**).

Classical monocytes (CD14^+^ monocytes) expressed *CD14*, *S100A9*, and *S100A8* (clusters 3 and 4) (**Figure 4D** and **Supplementary Figure S5C**). Non-classical monocytes (CD14^+^/CD16^+^ monocytes) expressed high levels of *FCGR3A*/CD16 (clusters 5 and 6). Cluster 0 expressed M1-associated genes (e.g., *IL-1B*, *TNF*, *HLA-DPB1*, and *IL2RA*), designated as M1-like macrophages, while cluster 1 expressed M2-associated genes (e.g., *CCL18*, *CTSD*, and *FN1*), consistent with an M2-like macrophage phenotype (**Figure 4D**). The final group, cluster 2 (marker-low cells), had relatively low expressions of the above-described marker genes.

There was notable heterogeneity between the monocytes and macrophages from the non-tumor thyroid tissue and those from different cancer stages (**Figure 4E**). Cells from the non-tumor thyroid tissue consisted predominantly of classical and non-classical monocytes, with rare M1- and M2-like macrophages. In contrast, cells from thyroid tumors were enriched with both M1- and M2-like macrophages. A previous study indicated that a disproportion of the pro-inflammatory M1-like and anti-inflammatory M2-like macrophages has been implicated in progressive disease stage (12). Our data revealed that an increased ratio of M2-like/M1-like macrophages was in accordance with the progressively advanced N stage (**Figures 4F, G**). Analysis of the survival data in TCGA cohort of patients with PTC revealed decreased OS in patients with the M1-low/M2-high signatures compared with those with the reversed phenotype of macrophages (*p* = 0.012) (**Figure 4H**). To determine the relationship between these cell subtypes and states, differentiation pseudotime trajectory analysis was performed using the Monocle 2 R package. Based on the ordering of pseudotime, CD14^+^ monocytes were divided into early (stage 1), middle (stage 2), and late (stage 4) stages, so were the CD14^+^/CD16^+^ monocytes from stage 5 to stage 2, and finally to stage 3 (**Figures 4I–K**). Along with the differentiation from early to late stages, M1- and M2-like macrophages accumulated in the middle and late stages (stages 2–4). In normal thyroid tissues, all the cells were confined to the early stage, while the proportions of myeloid cells in the middle and late stages continuously accumulated along with increasing disease N stage (**Figures 4L, M**).

### Myeloid Cells and T Cells Form Inhibitory Interactions in More Advanced Thyroid Cancer

**Figure 5:**
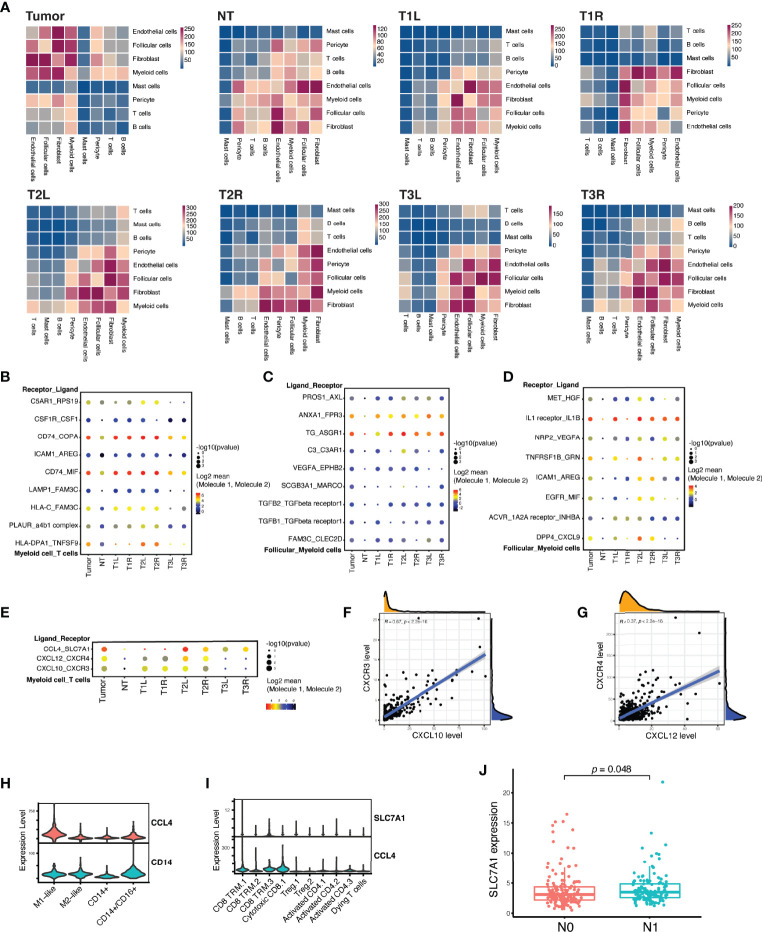
Myeloid cells and T cells formed inhibitory interactions in papillary thyroid carcinoma. **(A)** Heatmap of the number of significant ligand–receptor interactions among different cell lineages demonstrating a substantial heterogeneity between tumor and non-tumor (NT) thyroid samples, as well as between bilateral thyroid cancer samples from the sample patient. **(B)** Highlighted significant interactions between myeloid cells and T cells showing multiple interactions that promote M2-like macrophage polarization. **(C)** Highlighted significant interactions between myeloid cells and follicular cells showing multiple interactions that induce M2-like macrophage polarization. **(D)** Highlighted significant interactions between myeloid cells and follicular cells showing multiple interactions that drive tumor growth, motility, and metastasis. **(E)** Highlighted significant interactions between myeloid cells and T cells showing multiple interactions that recruit T cells, but dampen T-cell differentiation. **(F)** Strong linear correlation between the expressions of *CXCL10* and *CXCR3* in The Cancer Genome Atlas (TCGA) cohort of patients with PTC (*n* = 507). **(G)** Strong linear correlation between the expressions of *CXCL12* and *CXCR4* in TCGA cohort of patients with PTC (*n* = 507). **(H)** Violin plots showing the expression of *CCL4* in myeloid cells. **(I)** Violin plots showing the expression of *SLC7A1* in cytotoxic CD8 and CD8 TRM T cells. **(J)** A high expression of *SLC7A1* was associated with lymph node metastasis in TCGA cohort of patients with PTC (*n* = 281). *N0* indicates no regional lymph node metastasis, while *N1* indicates the presence of local lymph node metastasis.

CellPhoneDB v2.0 (17), a public repository of ligand–receptor interactions, was used to infer the cell–cell interactions among the different cell lineages (**Figure 5A**). Myeloid cells, fibroblasts, follicular cells, and endothelial cells were predicted to have a large number of cell–cell interactions (*p* < 0.01) (**Figure 5A**, upper left); conversely, B cells, T cells, pericytes, and mast cells had relatively fewer predicted interactions (**Figure 5A**, lower right). Myeloid cells, which are enriched in thyroid cancer, as described above, were predicted to have a high number of significant ligand–receptor interactions with other cell lineages, except for mast cells. The interaction patterns and magnitudes were slightly heterogenous across all tumor samples, even in paired samples from the same patient (**Figure 5A**).

We identified numerous biologically critical interactions between myeloid cells, T cells, and follicular cells, which were related to T-cell recruitment, M2-like macrophage polarization, malignant follicular cell progression, and T-cell inhibitory signaling. T cells expressed genes for ligands that induce M2-like macrophage polarization, including *CSF1* (binds to *CSF1R*) and *COPA* and *MIF* (both bind to *CD74*) (**Figure 5B**) (24, 25). Besides, malignant follicular cells also expressed genes for ligands that induce M2-like macrophage polarization, including *C3* (binds to *C3AR1*) and *ANAX1* (binds to *FPR3*) (**Figure 5C**) (26–28). In turn, myeloid cells expressed hepatocyte growth factor (HGF) that binds to the receptor (MET) in follicular cells (**Figure 5D**), activating the PI3K/Akt signaling pathway involved in cell growth, motility, and metastasis (29, 30). Myeloid cells expressed genes (*CXCL10* and *CXCL12*) for chemokines as chemoattractants to recruit T cells expressing *CXCR4* and/or *CXCR3* in T1 and T2 samples, but not in T3 (**Figure 5E**), which was in accordance with the histological assessment that indicated chronic lymphocytic thyroiditis in T1 and T2 samples (**Supplementary Figure S1**). The expression of *CXCL10* or *CXCL12* was strongly correlated with the expression of *CXCR3* or *CXCR4* in TCGA cohort of patients with PTC (**Figures 5F, G**). However, myeloid cells also expressed *CCL4*, specifically targeting cytotoxic CD8^+^ T and CD8^+^ TRM cells expressing *SLC7A1* (coding for the amino acid transporter) (**Figures 5E, H, I**), which could dampen the T-cell differentiation (31). Their interactions, as immunosuppressive signaling, were strongly associated with the presence of tumor and local lymph node metastasis in T2 and T3 samples (**Figure 5E**), which was confirmed in TCGA cohort, with the high expression of *SLC7A1* being significantly associated with lymph node metastasis (**Figure 5J**).

## Discussion

Building on prior work using scRNA-seq data to understand the cell-of-origin (32) and the TIME signature (33), we comprehensively characterized the cellular compartments and transcriptional phenotypes of PTC, revealing great heterogeneity in patients with bilateral PTC.

Genomic DNA sequencing revealed 2 pairs of bilateral tumors harboring the same *BRAF V600E* mutation, while the remaining pair of bilateral PTC had different genetic mutations, with *BRAF V600E* mutation in the right tumor and *RET/FARP1* fusion in the left tumor. *BRAF V600E* mutation, involved in activating the PI3K/Akt and MAPK signaling pathways, is the most common genetic alteration in adult PTC, while *RET* fusions (including *CCDC6*/*RET* and *NCOA4*/*RET*) are predominant in pediatric PTC (34). The *RET/FARP1* fusion was firstly identified in adult PTC with activation of the hedgehog signaling pathway in a T1L sample. The genetic alteration was consistent with the scRNA-seq analysis showing both PI3K/Akt and MAPK signaling pathways being enriched in PTC samples. However, the most predominantly enriched pathway in PTC was cytokine–cytokine receptor interaction, with 44 DEGs between the malignant and normal thyroid follicular cells. These malignant follicular cells expressed high levels of cytokines (*TNF* and *TNFSF15*), interleukins (*IL1A* and *IL18*), chemokines (*CXCL10*, *CXCL8*, and *CXCL9*), and other secreting factors (*GDF15* and *CSF1*) with distinctive roles in the TME. *IL1A* has been shown to inhibit the growth of thyroid carcinoma cells (35), whereas *GDF15* is involved in promoting thyroid cancer progression *via* the activation of *STAT3* (36). Moreover, *CSF1* induces pro-tumoral M2 macrophage polarization through binding to *CSF1R* (37), whereas *CXCL9* and *CXCL10* are involved in T-cell recruitment manifested as chronic lymphocytic thyroiditis (38), which has been associated with a better prognosis of PTC (39). Thus, we developed a 6-gene signature derived from the cytokine–cytokine receptor interaction pathway, which has been proven to be an independent predictor for the prognosis of patients with PTC. From the clinical perspective, dabrafenib, a specific BRAF kinase inhibitor, displayed only transient clinical benefit for *BRAF*-mutant thyroid cancer and finally became ineffective (40). Modulation of the highly activated cytokine–cytokine receptor interaction pathway may offer new therapeutic options to overcome dabrafenib-induced resistance in *BRAF*-mutated thyroid cancer. Collectively, our integrative analyses revealed great inter-tumor heterogeneity in bilateral PTC in the context of genetic alterations, copy number variations, and enriched signaling pathways, with important implications for adopting distinctive strategies for its management.

We observed that CD8 TRM-IFNG cells, consistent with a tissue-resident memory phenotype, as previously described (41), were diminished in more advanced disease. These cells expressed high levels of not only pro-inflammatory cytokines and chemokines (e.g., *IFNG*, *TNF*, *CCL4*, and *CCL5*) but also effector T-cell signatures, such as *PRF1* and *NKG7*, consistent with an antitumor phenotype. Previous studies have shown that the CD8 TRM-IFNG signature was a strong predictor for prolonged OS in patients with melanoma (41) and was enriched in the early disease stage of clear cell renal cell carcinoma (12). Further work is required to better understand its prognostic value in patients with PTC.

Through comprehensive analysis of the myeloid compartment, we observed an increase in anti-inflammatory M2-like macrophages and a decrease in pro-inflammatory M1-like macrophages in advanced PTC, which have been correlated with a worse prognosis of thyroid cancer (42, 43). In addition to canonical M2 markers (such as *CD163*, *FOLR2*, and *MS4A4A*), these M2-like macrophages expressed high levels of several cysteine cathepsins (*CTSD*, *CTSL*, and *CTSB*) and of the epithelial–mesenchymal transition (EMT) marker (*FN1*), which have previously been associated with tumor invasion and migration and have been implicated for potential targeted therapy (44–48). Multiple agents, including small-molecule inhibitors and neutralizing antibodies against *CSF1* or *CSF1R*, have been developed for targeting the *CSF1*/*CSF1R* axis to reprogram M2 macrophages (49, 50). However, their efficacy in treating solid tumors remains equivocal in clinical trials, probably due to the accompanying elevation of programmed death-ligand 1 (PD-L1) expression (51). Preclinical models have shown that the combination of targeting the *CSF1*/*CSF1R* axis with PD-L1/programmed cell death 1 (PD-1) blockade displayed synergistic antitumor effects in the treatment of several solid tumors (52, 53). Taken together, our findings provide a rationale to reprogram pro-tumoral M2-like macrophages together with PD-L1/PD-1 blockade in advanced thyroid cancer.

Myeloid cells coexisted with T cells across all samples, and we therefore computationally inferred intercellular communication between myeloid cells and the T-cell populations. This analysis identified several significant intercellular interactions between these two types of cells to cause immune dysfunction. Myeloid cells express chemokines (*CXCL12* and *CXCL10*) as chemoattractants to recruit T cells expressing their receptors (*CXCR4* and *CXCR3*), which is consistent with the pathological findings indicating chronic lymphocytic thyroiditis in P1 and P2 patients. Analysis of the PTC samples in TCGA database supported the strong linear correlation between the levels of these two chemokines and their corresponding receptors. The *CXCL12*/*CXCR4* axis also plays a critical role in the activation of oncogenic signaling networks and is associated with resistance to immune checkpoint inhibitors (ICIs) (54). Thus, modulating the *CXCL12*/*CXCR4* axis may exert antitumor effects and sensitize tumors resistant to ICI therapy. Myeloid cells, however, also express chemokine (*CCL4*) binding to the amino acid transporter SCL7A1, which was confirmed as an inhibitory signaling of effector T-cell differentiation (31). The recruited T cells within the TME produced factors (*CSF1*, *COPA*, and *MIF*) that induced the polarization of M2-like macrophages. Moreover, we also inferred cell–cell communication between follicular cells and myeloid cells. Our analysis indicated that malignant follicular cells also expressed ligands (*C3* and *ANAX1*) that encouraged the polarization of M2-like macrophages. In turn, myeloid cells expressed HGF that binds to the receptor (MET) in follicular cells, activating the PI3K/Akt signaling pathway involved in cell growth, motility, and metastasis (29, 30). Moreover, myeloid cells also expressed high levels of arginine (ARG) that binds to intercellular adhesion molecule 1 (ICAM1) in follicular cells, whose expression levels have been shown to be increased in PTC, poorly differentiated thyroid cancer (PDTC), and anaplastic thyroid cancer (ATC) and which has been proven to be an effective therapeutic target in advanced thyroid cancer (55, 56). Overall, these data support a model of cancer progression and localized immune dysfunction in PTC. Analysis of the cell–cell interactions in each sample revealed inter-tumor heterogeneity in terms of interaction intensity and pattern in bilateral PTC even with the same *BRAF V600E* mutation.

This study has several limitations. We included a relatively limited number of bilateral PTC samples subjected to scRNA-seq and genomic DNA sequencing. More samples are required to fully capture the inter-tumor heterogeneity in bilateral PTC. Additionally, more clinical and experimental effectors are required to determine the causality between myeloid cell and immune cell dysfunction and to establish the interactions between tumor cells and immune cells, which would provide guidance to reactivate antitumor immune response. Moreover, our study revealed that the heterogeneity of the TME was confined to well-differentiated PTC. Further work is required to comprehensively characterize tumors in more advanced stages with distant metastasis or poorly differentiated or anaplastic phenotype or radioiodine-refractory differentiated thyroid cancer.

## Conclusion

In conclusion, our study revealed great inter-tumor heterogeneity in the cellular composition and molecular phenotype of the TME in bilateral PTC. Prognostic enriched pathway signatures in malignant follicular cells, as well as the inhibitory interactions between myeloid cells and T cells, represent potential antitumor therapeutic targets, with the goal of offering assistance for precise diagnosis and treatment in bilateral PTC.

## Data Availability Statement

The scRNA-seq data have been deposited in the Gene Expression Omnibus (GEO) with accession number: GSE191288. R scripts for scRNA-seq analysis were available in GitHub with the following link: https://github.com/shenglei1988/scRNAseq-bilateral-PTC.

## Ethics Statement

The studies involving human participants were reviewed and approved by the ethics committees of Qilu Hospital of Shandong University and The Second Affiliated Hospital of Zhejiang University. The patients/participants provided written informed consent to participate in this study.

## Author Contributions

TW, LS, LL, and JS designed the study. LS and JS analyzed the bulk and single-cell RNA sequencing data. LCL, HZ, XFZ, YW, and XMZ contributed to biopsy samples and pathology analysis. TW, LS, and JS wrote the manuscript. All authors contributed to the article and approved the submitted version.

## Funding

The research was supported by grants from the National Natural Science Foundation of China (grant no. 81802642 to LS), the Medical and Health Technology Development Program of Shandong Province (grant no. 2017WS098 to TW) and the Zhejiang Provincial Natural Science Foundation of China (grant no. LY22H070002 to TW). The funders covered open-access publication fees, but were not involved in the research.

## Conflict of Interest

The authors declare that the research was conducted in the absence of any commercial or financial relationships that could be construed as a potential conflict of interest.

## Publisher’s Note

All claims expressed in this article are solely those of the authors and do not necessarily represent those of their affiliated organizations, or those of the publisher, the editors and the reviewers. Any product that may be evaluated in this article, or claim that may be made by its manufacturer, is not guaranteed or endorsed by the publisher.
