# Supplementary material for: Single-Cell Transcriptome Analysis Reveals Inter-Tumor Heterogeneity in Bilateral Papillary Thyroid Carcinoma
Source: Front Immunol. 2022 Apr 20;13:840811. doi: 10.3389/fimmu.2022.840811 (PMC9065345; doi:10.3389/fimmu.2022.840811)
Supplement: Supplementary file 1 [file DataSheet_1.pdf]

# *Supplementary Material*

**Supplementary Table S1. Enriched KEGG pathways in malignant thyroid follicular cells**

| ID | Description | GeneRatio | BgRatio | pvalue | p.adjust | qvalue |
|---|---|---|---|---|---|---|
| hsa04060 | Cytokine-cytokine receptor interaction | 44/458 | 295/8115 | 0.000 | 0.000 | 0.000 |
| hsa04151 | PI3K-Akt signaling pathway | 33/458 | 354/8115 | 0.003 | 0.028 | 0.023 |
| hsa04514 | Cell adhesion molecules | 29/458 | 149/8115 | 0.000 | 0.000 | 0.000 |
| hsa04510 | Focal adhesion | 29/458 | 201/8115 | 0.000 | 0.000 | 0.000 |
| hsa04010 | MAPK signaling pathway | 28/458 | 294/8115 | 0.004 | 0.035 | 0.029 |
| hsa05202 | Transcriptional misregulation in cancer | 27/458 | 193/8115 | 0.000 | 0.000 | 0.000 |
| hsa05205 | Proteoglycans in cancer | 27/458 | 205/8115 | 0.000 | 0.001 | 0.001 |
| hsa04062 | Chemokine signaling pathway | 23/458 | 192/8115 | 0.000 | 0.007 | 0.006 |
| hsa04061 | Viral protein interaction with cytokine and cytokine receptor | 22/458 | 100/8115 | 0.000 | 0.000 | 0.000 |
| hsa04015 | Rap1 signaling pathway | 22/458 | 210/8115 | 0.004 | 0.032 | 0.026 |
| hsa05417 | Lipid and atherosclerosis | 22/458 | 215/8115 | 0.005 | 0.036 | 0.029 |
| hsa04640 | Hematopoietic cell lineage | 20/458 | 99/8115 | 0.000 | 0.000 | 0.000 |
| hsa04668 | TNF signaling pathway | 20/458 | 112/8115 | 0.000 | 0.000 | 0.000 |
| hsa04512 | ECM-receptor interaction | 19/458 | 88/8115 | 0.000 | 0.000 | 0.000 |
| hsa05323 | Rheumatoid arthritis | 19/458 | 93/8115 | 0.000 | 0.000 | 0.000 |
| hsa04145 | Phagosome | 19/458 | 152/8115 | 0.001 | 0.010 | 0.008 |
| hsa04610 | Complement and coagulation cascades | 18/458 | 85/8115 | 0.000 | 0.000 | 0.000 |
| hsa05418 | Fluid shear stress and atherosclerosis | 18/458 | 139/8115 | 0.001 | 0.010 | 0.008 |
| hsa04933 | AGE-RAGE signaling pathway in diabetic complications | 17/458 | 100/8115 | 0.000 | 0.001 | 0.001 |
| hsa04064 | NF-kappa B signaling pathway | 17/458 | 104/8115 | 0.000 | 0.001 | 0.001 |
| hsa05410 | Hypertrophic cardiomyopathy | 16/458 | 90/8115 | 0.000 | 0.001 | 0.001 |

| | | | | | | |
|---|---|---|---|---|---|---|
| hsa04670 | Leukocyte transendothelial migration | 16/458 | 114/8115 | 0.001 | 0.008 | 0.007 |
| hsa04270 | Vascular smooth muscle contraction | 16/458 | 134/8115 | 0.003 | 0.032 | 0.025 |
| hsa05144 | Malaria | 15/458 | 50/8115 | 0.000 | 0.000 | 0.000 |
| hsa05414 | Dilated cardiomyopathy | 15/458 | 96/8115 | 0.000 | 0.005 | 0.004 |
| hsa05142 | Chagas disease | 15/458 | 102/8115 | 0.001 | 0.008 | 0.006 |
| hsa05146 | Amoebiasis | 15/458 | 102/8115 | 0.001 | 0.008 | 0.006 |
| hsa05150 | Staphylococcus aureus infection | 14/458 | 96/8115 | 0.001 | 0.010 | 0.008 |
| hsa04350 | TGF-beta signaling pathway | 13/458 | 94/8115 | 0.002 | 0.022 | 0.018 |
| hsa04928 | Parathyroid hormone synthesis, secretion and action | 13/458 | 106/8115 | 0.006 | 0.046 | 0.037 |
| hsa04918 | Thyroid hormone synthesis | 12/458 | 75/8115 | 0.001 | 0.010 | 0.008 |
| hsa05133 | Pertussis | 12/458 | 76/8115 | 0.001 | 0.011 | 0.009 |
| hsa05140 | Leishmaniasis | 12/458 | 77/8115 | 0.001 | 0.012 | 0.010 |
| hsa05032 | Morphine addiction | 12/458 | 91/8115 | 0.005 | 0.036 | 0.029 |
| hsa04657 | IL-17 signaling pathway | 12/458 | 94/8115 | 0.006 | 0.046 | 0.037 |
| hsa05143 | African trypanosomiasis | 11/458 | 37/8115 | 0.000 | 0.000 | 0.000 |
| hsa04978 | Mineral absorption | 11/458 | 60/8115 | 0.000 | 0.007 | 0.006 |
| hsa05412 | Arrhythmogenic right ventricular cardiomyopathy | 11/458 | 77/8115 | 0.004 | 0.032 | 0.026 |
| hsa05219 | Bladder cancer | 10/458 | 41/8115 | 0.000 | 0.001 | 0.001 |
| hsa05321 | Inflammatory bowel disease | 10/458 | 65/8115 | 0.003 | 0.030 | 0.024 |
| hsa05134 | Legionellosis | 9/458 | 57/8115 | 0.004 | 0.035 | 0.029 |
| hsa05340 | Primary immunodeficiency | 7/458 | 38/8115 | 0.005 | 0.036 | 0.029 |

**Supplementary Table S2. 44 of differentially expressed genes in cytokine-cytokine receptor interaction between malignant thyroid follicular cells and non-tumor follicular cells**

| Entrez ID | Gene symbol | p_val | avg_log2FC | pct.1 | pct.2 | p_val_adj |
|---|---|---|---|---|---|---|
| 3627 | CXCL10 | 0.000 | 384.504 | 0.628 | 0.944 | 0.000 |
| 9518 | GDF15 | 0.000 | 338.986 | 0.583 | 0.821 | 0.000 |
| 3576 | CXCL8 | 0.000 | 264.674 | 0.313 | 0.436 | 0.000 |
| 4283 | CXCL9 | 0.000 | 221.787 | 0.513 | 0.726 | 0.000 |
| 2921 | **CXCL3** | 0.000 | 159.369 | 0.626 | 0.811 | 0.000 |
| 9235 | IL32 | 0.000 | 96.989 | 0.851 | 0.963 | 0.000 |
| 6351 | CCL4 | 0.000 | 86.455 | 0.925 | 0.997 | 0.000 |
| 9560 | CCL4L2 | 0.000 | 86.455 | 0.925 | 0.997 | 0.000 |
| 6364 | CCL20 | 0.000 | 77.821 | 0.794 | 0.922 | 0.000 |
| 6348 | CCL3 | 0.000 | 74.304 | 0.854 | 0.964 | 0.000 |
| 6349 | CCL3L1 | 0.000 | 74.304 | 0.854 | 0.964 | 0.000 |
| 4050 | LTB | 0.000 | 67.271 | 0.822 | 0.988 | 0.000 |
| 3552 | **IL1A** | 0.000 | 62.628 | 0.552 | 0.766 | 0.000 |
| 8793 | TNFRSF10D | 0.000 | 53.046 | 0.551 | 0.862 | 0.000 |
| 9966 | TNFSF15 | 0.000 | 49.338 | 0.514 | 0.790 | 0.000 |
| 6352 | **CCL5** | 0.000 | 38.396 | 0.916 | 1.000 | 0.000 |
| 6376 | CX3CL1 | 0.000 | 29.855 | 0.578 | 0.720 | 0.000 |
| 8743 | TNFSF10 | 0.000 | 29.559 | 0.741 | 0.737 | 0.000 |
| 3625 | INHBB | 0.000 | 29.300 | 0.886 | 0.994 | 0.001 |
| 970 | CD70 | 0.000 | 22.921 | 0.537 | 0.903 | 0.000 |
| 3575 | IL7R | 0.000 | 21.357 | 0.810 | 0.983 | 0.000 |
| 7852 | CXCR4 | 0.000 | 20.233 | 0.931 | 0.995 | 0.000 |
| 7124 | TNF | 0.000 | 13.554 | 0.867 | 0.882 | 0.000 |
| 51330 | **TNFRSF12A** | 0.000 | 13.554 | 0.867 | 0.882 | 0.000 |

| | | | | | | |
|---|---|---|---|---|---|---|
| 3557 | IL1RN | 0.000 | 13.202 | 0.616 | 0.803 | 0.000 |
| 650 | BMP2 | 0.000 | 12.471 | 0.663 | 0.550 | 0.000 |
| 7042 | TGFB2 | 0.000 | 12.408 | 0.421 | 0.493 | 0.000 |
| 3595 | IL12RB2 | 0.000 | 12.268 | 0.564 | 0.942 | 0.000 |
| 7133 | TNFRSF1B | 0.000 | 10.789 | 0.778 | 0.997 | 0.000 |
| 284340 | CXCL17 | 0.000 | 9.886 | 0.528 | 0.670 | 0.000 |
| 2919 | **CXCL1** | 0.000 | 9.886 | 0.528 | 0.670 | 0.000 |
| 3606 | **IL18** | 0.000 | 9.032 | 0.477 | 0.617 | 0.000 |
| 7293 | TNFRSF4 | 0.000 | 7.023 | 0.859 | 0.986 | 0.000 |
| 3559 | IL2RA | 0.000 | 6.200 | 0.824 | 0.993 | 0.000 |
| 58191 | CXCL16 | 0.000 | 5.850 | 0.697 | 0.887 | 0.000 |
| 3976 | LIF | 0.000 | 4.528 | 0.504 | 0.642 | 0.000 |
| 654 | BMP6 | 0.000 | 4.288 | 0.539 | 0.886 | 0.000 |
| 6846 | XCL2 | 0.000 | 2.980 | 0.858 | 0.986 | 0.000 |
| 6367 | CCL22 | 0.000 | 2.545 | 0.445 | 0.658 | 0.000 |
| 655 | BMP7 | 0.000 | 1.656 | 0.702 | 0.628 | 0.000 |
| 8784 | TNFRSF18 | 0.000 | 1.606 | 0.805 | 0.999 | 0.000 |
| 7048 | TGFBR2 | 0.000 | -1.014 | 0.917 | 0.987 | 0.000 |
| 6358 | CCL14 | 0.000 | -2.887 | 0.699 | 0.195 | 0.000 |
| 3977 | LIFR | 0.000 | -5.561 | 0.696 | 0.427 | 0.000 |

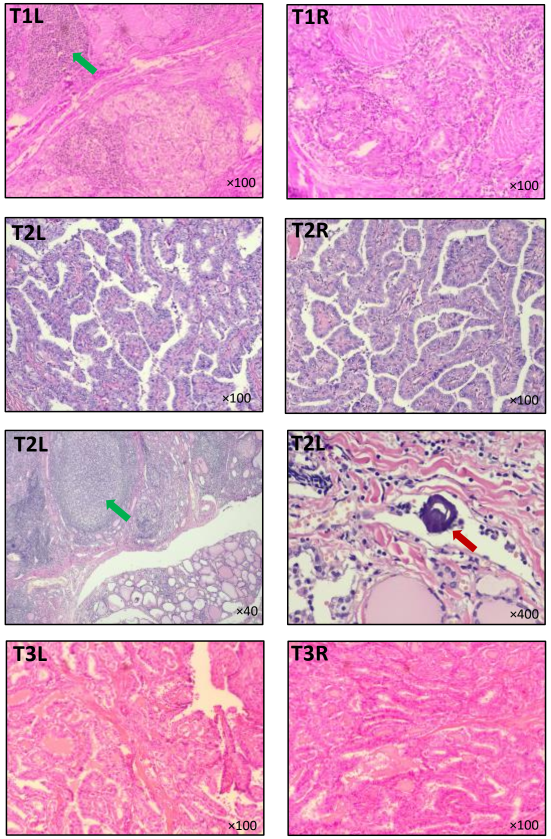


Supplementary Figure S1. Histological assessment of papillary thyroid carcinoma (PTC) in each patient. T1L and T1R: PTC from the left lobe and right lobe of the patient P1, respectively. T2L and T2R: PTC from the left lobe and right lobe of the patient P2, respectively. T3L and T3R: PTC from the left lobe and right lobe of the patient P3, respectively. Green arrow shows lymphocyte infiltration in thyroid tissue, indicative of chronic lymphocytic thyroiditis (CLT). Red arrow shows psammoma body, indicative of intrathyroidal spreading.

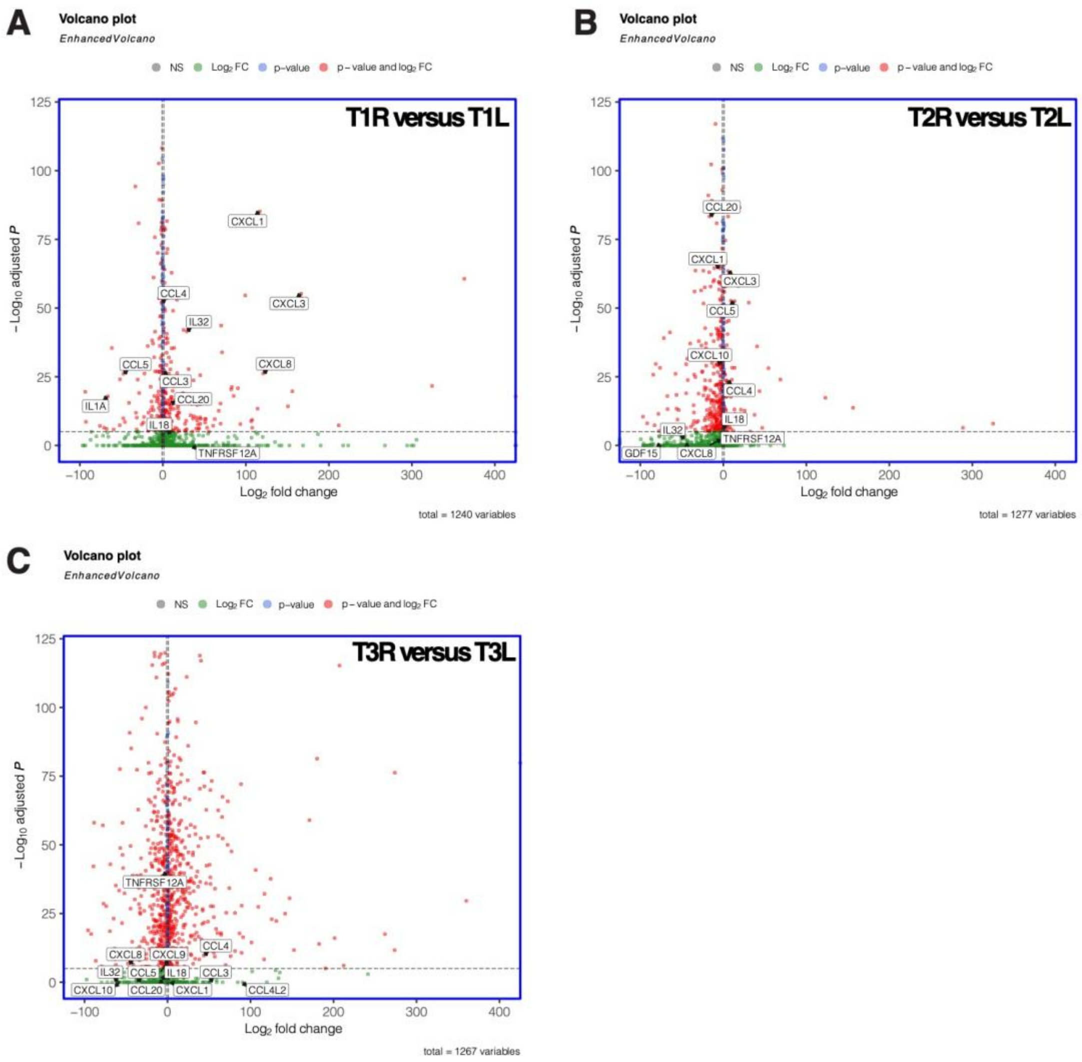


Supplementary Figure S2. Differentially expressed genes between the right and left tumor in patient by patient. T1L and T1R: PTC from the left lobe and right lobe of the patient P1, respectively. T2L and T2R: PTC from the left lobe and right lobe of the patient P2, respectively. T3L and T3R: PTC from the left lobe and right lobe of the patient P3, respectively.

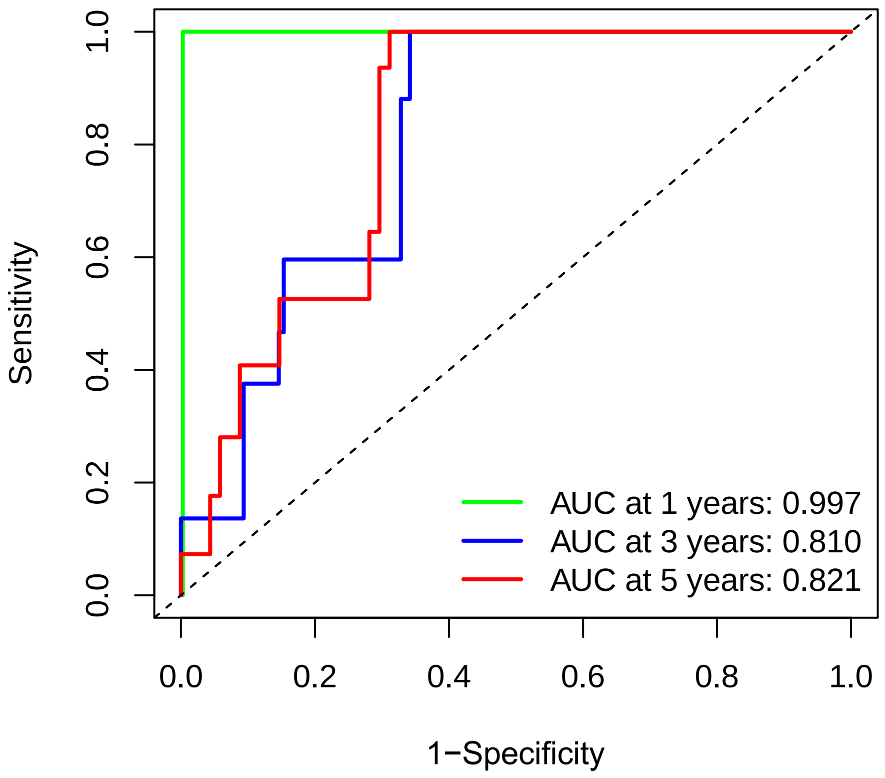


Supplementary Figure S3. Predicting capability of the 6-gene signature for overall survival in patients with PTC. AUC: area under the curve.

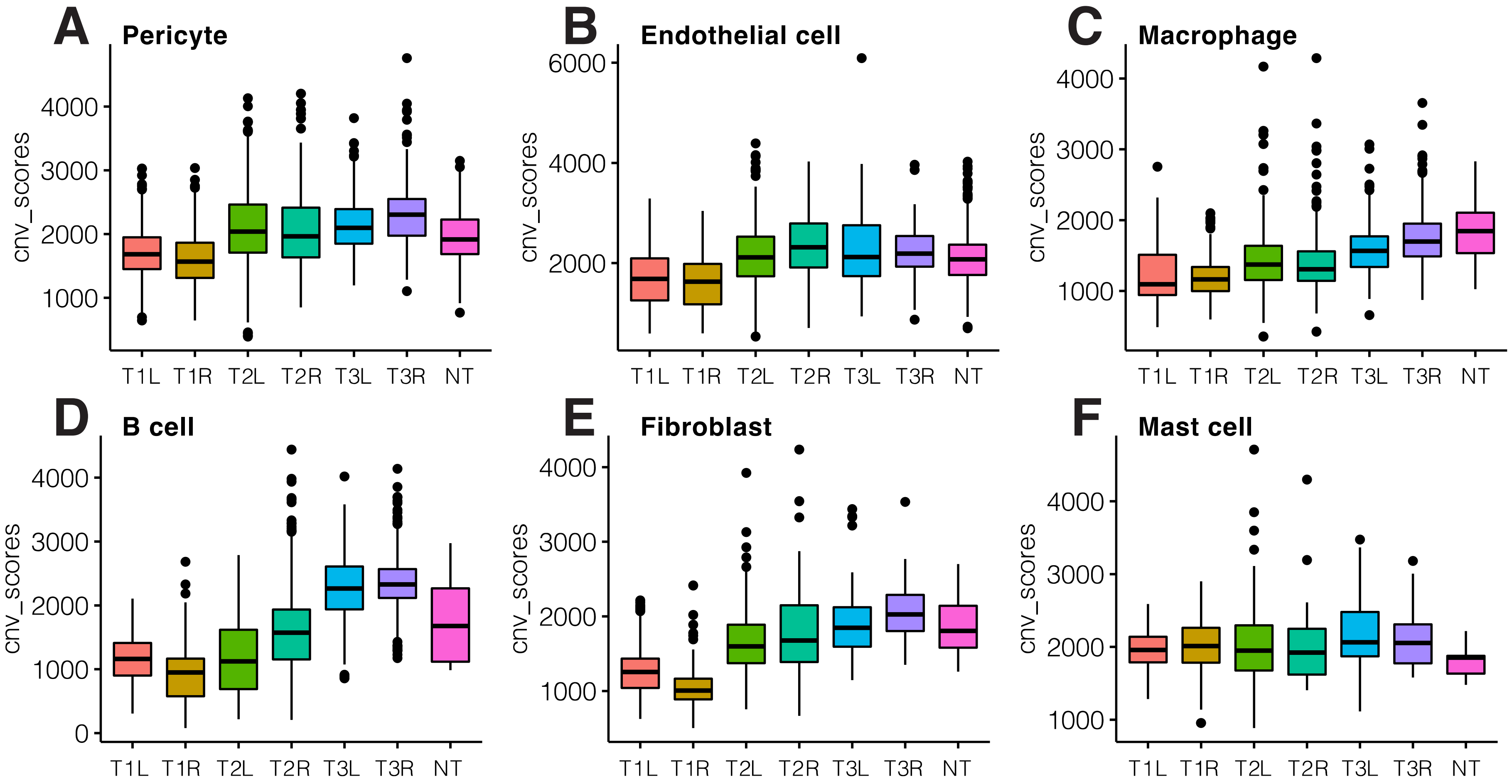


Supplementary Figure S4. Comparison of copy number variation (CNV) scores of each cell lineage in different thyroid samples.

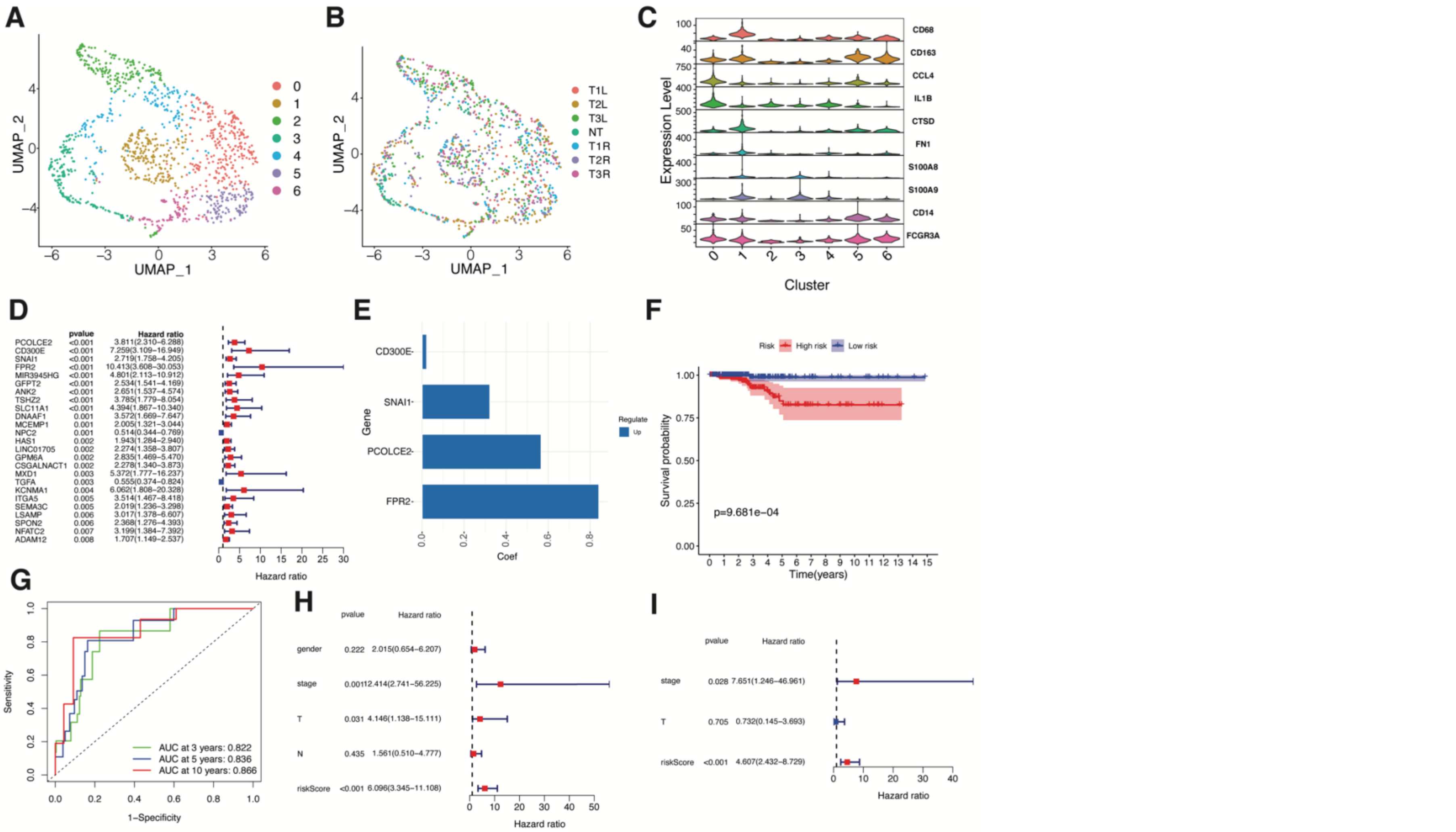


Supplementary Figure S5. Prognostic significance of a gene signature of myeloid cells in papillary thyroid cancer. (A) UMAP plot of the classification of myeloid cells (n=1,179) from malignant and non-malignant thyroid tissue in an unsupervised clustering approach. (B) UMAP plot of myeloid cells in different thyroid samples. (C) Violin plot showing the expression distribution of canonical marker genes in each cell cluster. (D) Prognostic value of differentially expressed genes identified in myeloid cells in malignant thyroid tissue as compared to normal

tissue. (E) Coefficient of 4 genes for constructing a multi-gene signature. (F) Kaplan-Meier curve of overall survival in patients from the TCGA PTC cohort group by low and high risk score of 4-gene signature identified in tumor-associated macrophages. (G) Predicting capability of the 4-gene signature for overall survival in patients with PTC. AUC: area under the curve. (H) and (I) Unadjusted and adjusted prognostic significance of 4-gene signature and other clinicopathological factors for overall survival in patients with PTC.
